# Achieving Predictable Esthetics With Early Implant Placement and Soft Tissue Modeling: A Case Report and Literature Review

**DOI:** 10.7759/cureus.64549

**Published:** 2024-07-15

**Authors:** Swapnali Mhatre, Richa Gupta, Mirella Vaz, Dheeraj Bijlani, Mridula Joshi, Prachi Gholap, Uttam Shetty, Reema Srichand

**Affiliations:** 1 Prosthodontics, Crown and Bridge, and Implantology, Bharati Vidyapeeth (Deemed to be University) Dental College and Hospital, Navi Mumbai, IND; 2 Prosthodontics, Bharati Vidyapeeth (Deemed to be University) Dental College and Hospital, Navi Mumbai, IND

**Keywords:** white esthetic, pink esthetic, anterior esthetic, implant stability, customized healing abutment, soft tissue modeling, early implant placement

## Abstract

In the modern era, patients are increasingly concerned about dental esthetics. Edentulism can significantly impact the appearance, occlusion, and self-esteem of the patient. Treatment options like removable dentures, fixed crown and bridge prostheses, and resin-retained bridges are available to replace missing teeth. Implant therapy is popular due to its high success rates and long-term durability. However, its efficacy can be compromised by errors in treatment planning, surgery, tissue care, and infections. Thus, meticulous planning and execution are crucial. Clinicians must have the expertise to manage difficulties during surgery and maintain stable soft tissue. The stability of tissues around osseointegrated implants affects long-term clinical stability and esthetics. Early implant placement is recommended for missing teeth in the esthetic zone, aiming to place implants in anatomically and functionally correct positions for durable and esthetic results. Patients with high cosmetic demands, thin gingival biotypes, and high smile lines pose challenges. Assessing Pink and White esthetics guides treatment planning. Advantages of early implant placement include simplified procedures and reduced post-surgical complications. Soft tissue molding is achieved using customized healing abutments and temporizing with fiber-reinforced resin-bonded prostheses. Customized healing abutments preserve socket width, prevent soft tissue collapse, and promote natural contouring, eliminating the need for secondary surgeries and aiding spontaneous healing. This case report outlines a comprehensive approach to achieving predictable esthetics through early implant placement and soft tissue modeling in a patient with a traumatically avulsed maxillary central incisor.

## Introduction

To replace a missing tooth in the esthetic region with an implant presents a significant challenge to dentists due to the high expectations for both functional and esthetic outcomes. The high clinical success rates associated with implant therapy have encouraged practitioners to explore various implant placement protocols in esthetic zones [1]. These protocols can be used depending on individual patient's needs and the specific characteristics of the implant site.

According to the classification based on the healing of soft tissue, implant placement is categorized into four types. Type 1 involves immediate implant placement in the socket immediately after extraction [2]. This protocol requires careful consideration of factors such as socket morphology and primary stability to ensure successful outcomes. Type 2 entails placing an implant after significant soft tissue healing has occurred but before bone fill has taken place. This approach allows for adequate soft tissue maturation before implant placement, promoting better esthetic outcomes. Type 3 involves implant placement in a site where a significant amount of bone fill has taken place both clinically and radiographically. This protocol is suitable for patients with delayed implant placement needs, ensuring optimal bone support for long-term stability. Type 4 comprises implant placement in fully healed bone, offering a stable foundation for implant-supported restorations. This protocol is typically employed in cases where the extraction site has fully healed, minimizing the risk of complications associated with early implant placement. By carefully selecting the appropriate implant placement protocol based on the patient's individual characteristics and clinical considerations, dentists can achieve predictable outcomes in esthetic zone implant therapy. However, thorough preoperative assessment and meticulous surgical technique are essential to optimize success rates and meet patient expectations for both function and esthetics.

According to the International Team for Implantology (ITI) Consensus Conference 2018 on implant placement protocols, the immediate implant placement approach is characterized by its immediacy, aiming to minimize the time between extraction and implantation. Immediate implant placement is often chosen when there is adequate primary stability and favorable socket morphology, allowing for successful integration of the implant [3]. Early implant placement, on the other hand, occurs during the soft tissue healing phase, typically between four to eight weeks after tooth extraction. Alternatively, it may occur during partial bone healing, approximately 12-16 weeks post extraction. This protocol allows for sufficient time for soft tissue maturation and early bone healing before implant placement. Early implant placement is selected based on clinical assessment and diagnostic considerations, aiming to achieve optimal conditions for successful osseointegration and soft tissue integration. Late implant placement involves implant placement after the bone has completely healed, typically six months or more following extraction. This protocol allows for ample time for complete bone regeneration and maturation, ensuring a stable foundation for implant placement. Late implant placement is advocated in patients with compromised bone quality or quantity requiring additional time for bone regeneration. Each implant placement protocol has specific indications based on the individual patient's diagnosis, the available bone and soft tissue, as well as other clinical factors. The choice of protocol is guided by careful preoperative assessment and is case-dependent.

According to Misch, implant placement timing is categorized into four main types: immediate, early, delayed, and late [4]. Immediate placement involves placing the implant concurrently with tooth extraction, maximizing efficiency by reducing the number of surgical procedures needed. Early placement occurs within four to six weeks after extraction, allowing for initial healing to take place while still benefiting from preserved bone volume. Delayed placement, on the other hand, takes place three to four months post extraction, providing more time for complete healing and bone remodeling before implant placement. Late placement, designated for instances beyond four months post extraction, may be necessary due to various factors such as additional bone augmentation requirements or patient preferences.

In its Consensus Conference 2013, the ITI clearly indicated the most prevailing trend in dental implant placement protocols with the focus shifted towards selecting immediate or early placement protocols [5]. This shift was driven by several factors, notably the understanding of post-extraction alveolar remodeling [6,7]. Immediate and early implant placement protocols offer distinct advantages, including shorter treatment time, preservation of soft tissue morphology, and improved esthetics. But at the same time, it also includes the risk of mucosal recession and the requirement of a skilled operator [8]. Guided by cone-beam computed tomography (CBCT) findings, they also recommended when to use each treatment option, facilitating precision in treatment planning. Immediate implant placement is indicated in regions where facial wall bone is fully intact with more than 1 mm thickness and a thick biotype of the gingiva. Early implant placement is indicated when local bone structure permits correct three-dimensional implant positioning and sufficient primary stability. It promotes soft tissue recovery within four to eight weeks and is also advised for areas with thin facial walls or compromised sites. This strategy is commonly employed in anterior maxillary extractions [9]. It yields favorable regenerative and esthetic outcomes with high predictability and minimal risk of recession. However, delayed placement necessitates opening the flap after soft tissue healing to facilitate contour augmentation through guided bone regeneration, involving a second surgical intervention [10]. The primary objective of anterior zone implants is to attain superior esthetic outcomes with a high level of predictability and minimal occurrence of complications [11].

Long-term success in the esthetic zone with implants following tooth extraction mainly depends on the stability of peri-implant tissues. Secondary objectives encompass minimizing surgical interventions, particularly open flap procedures, as well as reducing patient discomfort and shortening the overall healing and treatment duration [12]. To achieve this, a three-dimensional approach is of vital importance for implant placement: mesial-distal, buccal-palatal, and occlusal-apical.

Mesial-distal is recommended to place the maxillary central incisor slightly distal to the midline of the tooth to avoid impinging the nerve, as the nasopalatine foramina commonly dictates distal placement [13]. Thus, in order to preserve the bone and retain the papillae, the clinician must place the implant 1.5-2.0 mm from the tooth. For buccal-palatal, the ideal implant placement is slightly palatal to the palatoincisal line angle which creates a buccal emergence profile. If the implant is angled palatal to this line, a ridge lap restoration may be necessary. On the other hand, angling the implant too labially will lead to a loss of labial gingival height and an uneven gingival margin. In occlusal-apical, the ideal position is 3-4 mm from the “anticipated” dento-gingival junction which provides room to develop an emergence profile. Deeply placed implants are difficult to clean and debris or excess cement may get entrapped while shallow implants are at risk of exposure and may not allow adequate space for the prosthesis.

Implant treatment success depends greatly on stability as well as the architecture of the peri-implant tissues, which are influenced by various factors. These include the nature of the surgical procedure undertaken, the integrity and volume of the soft tissue, as well as the prosthesis and the abutment design. Several studies support the importance of these factors in peri-implant health. Schwarz et al. reported that the use of guided bone regeneration techniques during implant placement significantly influences soft tissue stability and marginal bone levels around implants [14]. Similarly, a study by Linkevicius et al. emphasized the role of adequate soft tissue thickness and quality in preventing complications such as mucosal recession and peri-implant inflammation [15]. As alveolar ridge resorption occurs after tooth extraction, there is a gradual reduction of not only bone but also soft tissue volume. This resorption process is leading to the formation of recession defects[16]. The severity and characteristics of these defects depend upon various factors, including the timing of dental implant placement.

Insufficient attached gingiva surrounding dental implants poses significant clinical challenges, manifesting as an accumulation of plaque and gingival inflammation. Other signs are recession and discomfort during dental hygiene practices. To mitigate these issues, soft tissue grafting procedures are frequently employed to augment attached mucosa [14]. Augmentation of mucosal volume serves not only esthetic purposes but also crucial functional roles, including recession prevention, facilitation of dental hygiene practices, and the preservation of marginal bone and overall periodontal health [17].

Soft tissue grafting procedures are indicated in deficiencies in mucosal thickness surrounding dental implants. In cases where there is inadequate attached gingiva, the preferred treatment plan involves a gingival flap or vestibuloplasty, either alone or in combination with a graft. Among grafting options available, autogenous transplants such as free gingival or subepithelial tissue grafts are widely regarded as the gold standard due to their excellent biocompatibility and predictable outcomes. Alternative therapeutic modalities include the use of allogenic dermal matrix grafts or collagen matrices in conjunction with the apically positioned flap or vestibuloplasty. These materials offer viable options for soft tissue augmentation, providing structural support and promoting tissue regeneration. By augmenting attached tissue volume, these procedures contribute to the improvement of soft tissue quality, thereby reducing the risk of complications such as tissue dehiscences. Combining abutment implant connection and soft tissue grafting procedures have demonstrated efficacy in augmenting soft tissue volume and minimizing the necessity for additional interventions. Gingival advancement flap in conjunction with a subepithelial tissue augmentation has emerged as a dependable treatment modality during the second stage of implant surgery [18]. This approach offers a reliable means to enhance soft tissue volume and ensure optimal peri-implant mucosal health [19]. However, soft tissue augmentation surgery is preferred either before implant placement or during the healing period over simultaneous procedures. Precaution needs to be taken due to the challenges associated with stabilizing the graft during simultaneous implant placement, which may compromise the success of both procedures.

Implant healing abutments serve a dual purpose in the implant healing process. Firstly, they facilitate peri-implant tissue healing during the initial phases post-implantation, aiding in the contouring of soft tissue present surrounding the dental implant. Secondly, they act as a barrier, preventing the accumulation of plaque or debris at the implant site. Healing abutments are typically classified into two categories: standard and customized. Standard healing abutments are prefabricated cylindrical structures, non-hex type, designed for easy insertion in any direction [20]. Customized healing abutments are tailored to individual patient needs, offering a natural appearance and promoting a favorable emergence profile. These customized abutments are modified to guide the gingival collar into the desired shape, thus contributing to achieving esthetically pleasing outcomes. Thus, the prerequisite for an esthetically successful outcome is meticulous presurgical planning. This clinical report illustrates early implant placement and soft tissue modeling in order to achieve predictable esthetics.

## Case presentation

**Figure 1:**
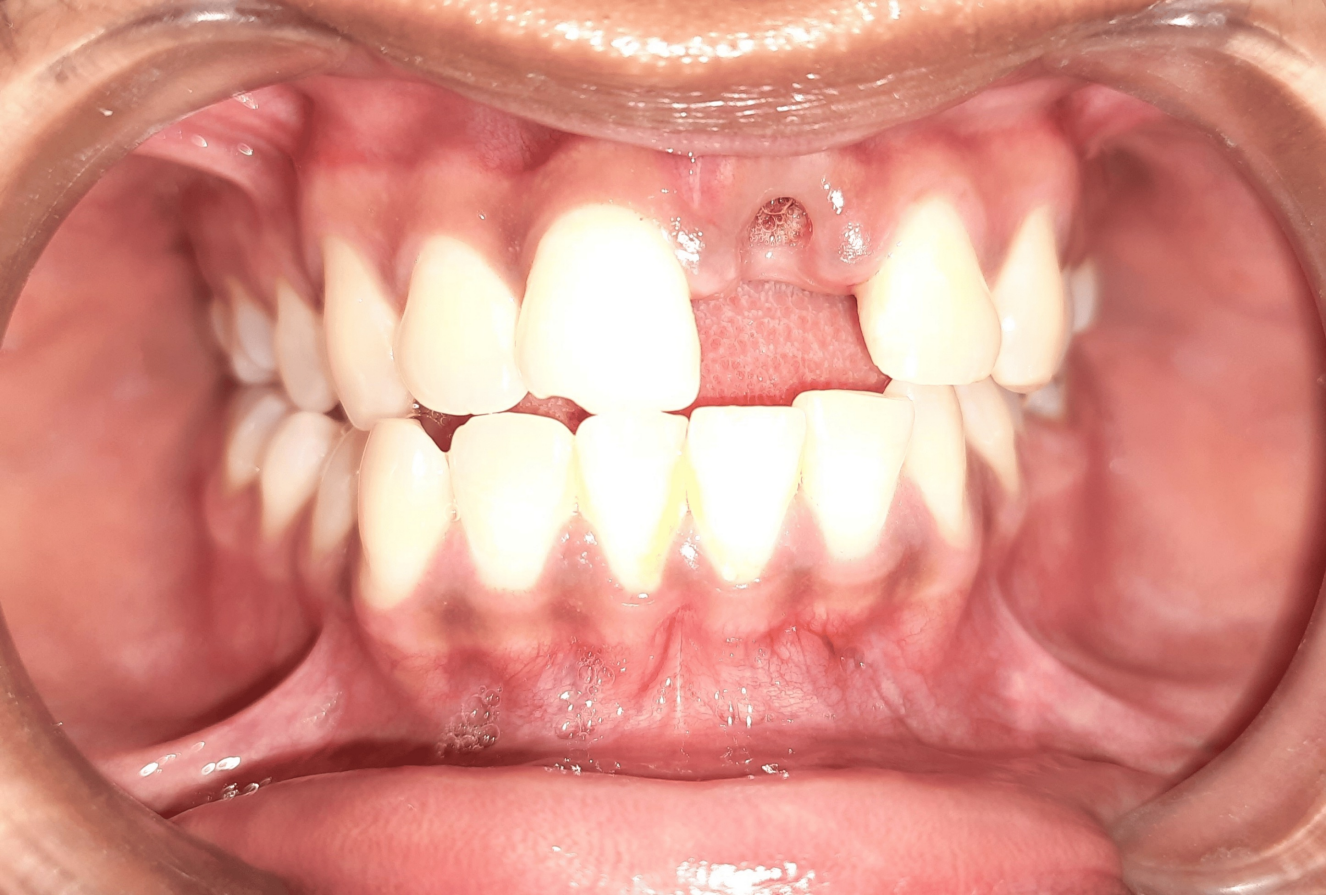
Preoperative intraoral view of edentulous area

**Figure 2:**
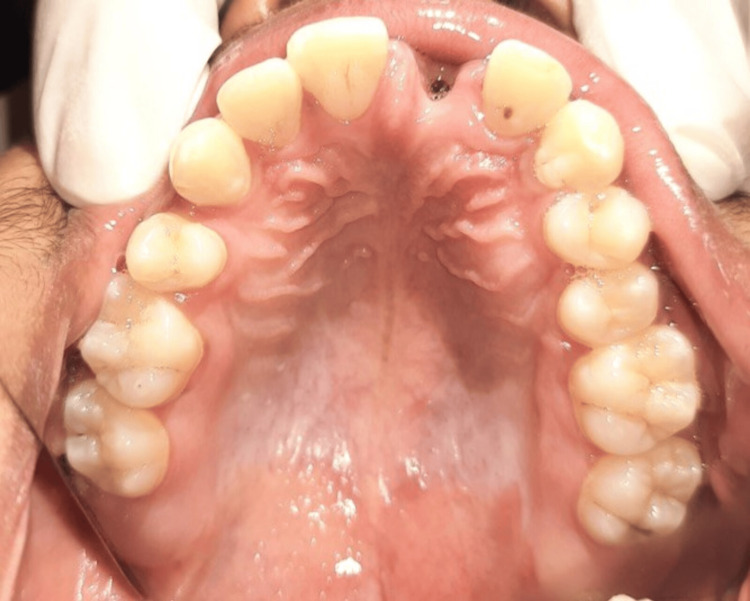
Preoperative occlusal view showing complete avulsion of tooth

A 25-year-old male presented to the Department of Prosthodontics with the chief complaint of a missing tooth in the upper anterior region following trauma sustained two days prior (Figure 1). The patient reported an accidental fall into a swimming pool, resulting in the avulsion of the left maxillary central incisor, which was lost in the pool. Apart from this dental trauma, the patient was in good health, with no apparent extraoral or intraoral injuries. He expressed a strong desire for a high-quality cosmetic solution and declined the option of having his adjacent teeth prepared for a traditional three-unit fixed dental prosthesis. On clinical examination, the patient's smile line was observed during functional movements, such as talking or smiling, revealing the interdental papilla. He exhibited a low lip line and a Class II facial profile. After discussing various treatment modalities with the patient, he refused the conventional prosthetic option, so the treatment plan involving dental implant placement followed by the crown was planned (Figure 2). 

The assessment of gingival biotype played an important role in evaluating peri-implant tissues during the implant therapy process. Utilizing a Williams probe, peri-implant tissues were meticulously examined to identify any signs of inflammation and to determine the peri mucosal depth. A periodontal probe was used to record the Gingival biotype and was classified as thick. Esthetic assessment was conducted to ensure a comprehensive evaluation of both Pink and White esthetics. The Pink esthetics evaluation focused on critical aspects such as mesial and distal interdental papilla morphology, level of the soft tissue, color, texture, contour, and alveolar process deficiency. Each element was recorded meticulously to gauge its contribution to the overall soft tissue esthetics surrounding the implant site. The White esthetics evaluation involved a detailed appraisal of tooth-related parameters, including the form of the tooth, contour, color (hue and value), the surface texture of the tooth, and its translucency. Special emphasis was placed on comparing these parameters with those of the contralateral reference tooth, ensuring a balanced and harmonious appearance between the implant restoration and its natural tooth counterpart. Detailed intraoral examination revealed missing 21 and Elli’s Class 1 fracture with 11. Radiographically, intact labial and palatal bone plates were evident.

**Figure 3:**
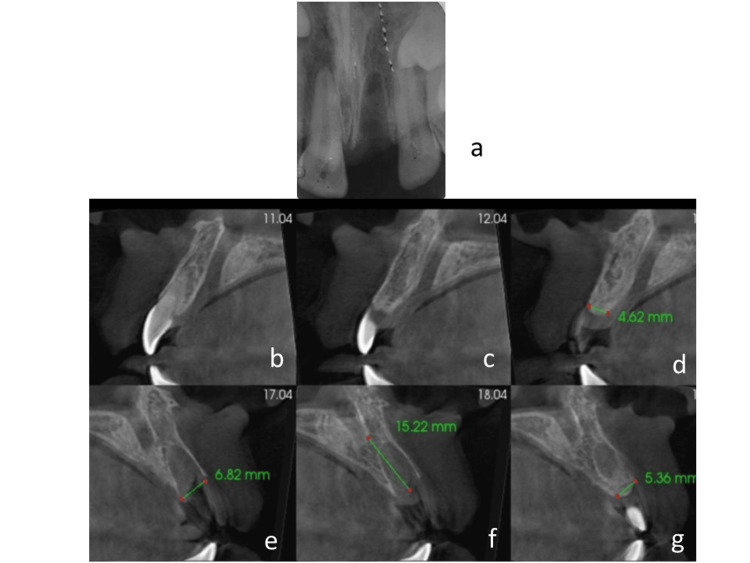
Radiographic investigation (a) Periapical view of the edentulous area;  (b)-(g) CBCT of the cross-sectional view of intact buccal and lingual bone plate showing the thickness of available bone CBCT: cone-beam computed tomography

Based on the diagnostic findings, he was categorized as an American College of Prosthodontists (ACP) Prosthodontic Diagnostic Index (PDI) Class I partially edentulous patient. The edentulous area was confined to a single arch involving the maxillary anterior region, not involving more than two incisors. The occlusion was ideal and minimally compromised with no need for preprosthetic surgery. The residual ridge morphology suggested class 1 edentulism. Class I partially edentulous patients typically exhibit favorable periodontal conditions, adequate remaining dentition, and minimal occlusal discrepancies, allowing for relatively straightforward treatment planning and prosthetic interventions. It was classified as advanced according to SAC (Straightforward, Advanced, Complex) classification by ITI since it involved a single tooth in the esthetic zone. Radiographic evaluation was suggestive of intact bony plates, adequate bone height, and absence of any pathology or fracture from trauma (Figure 3).

**Figure 4:**
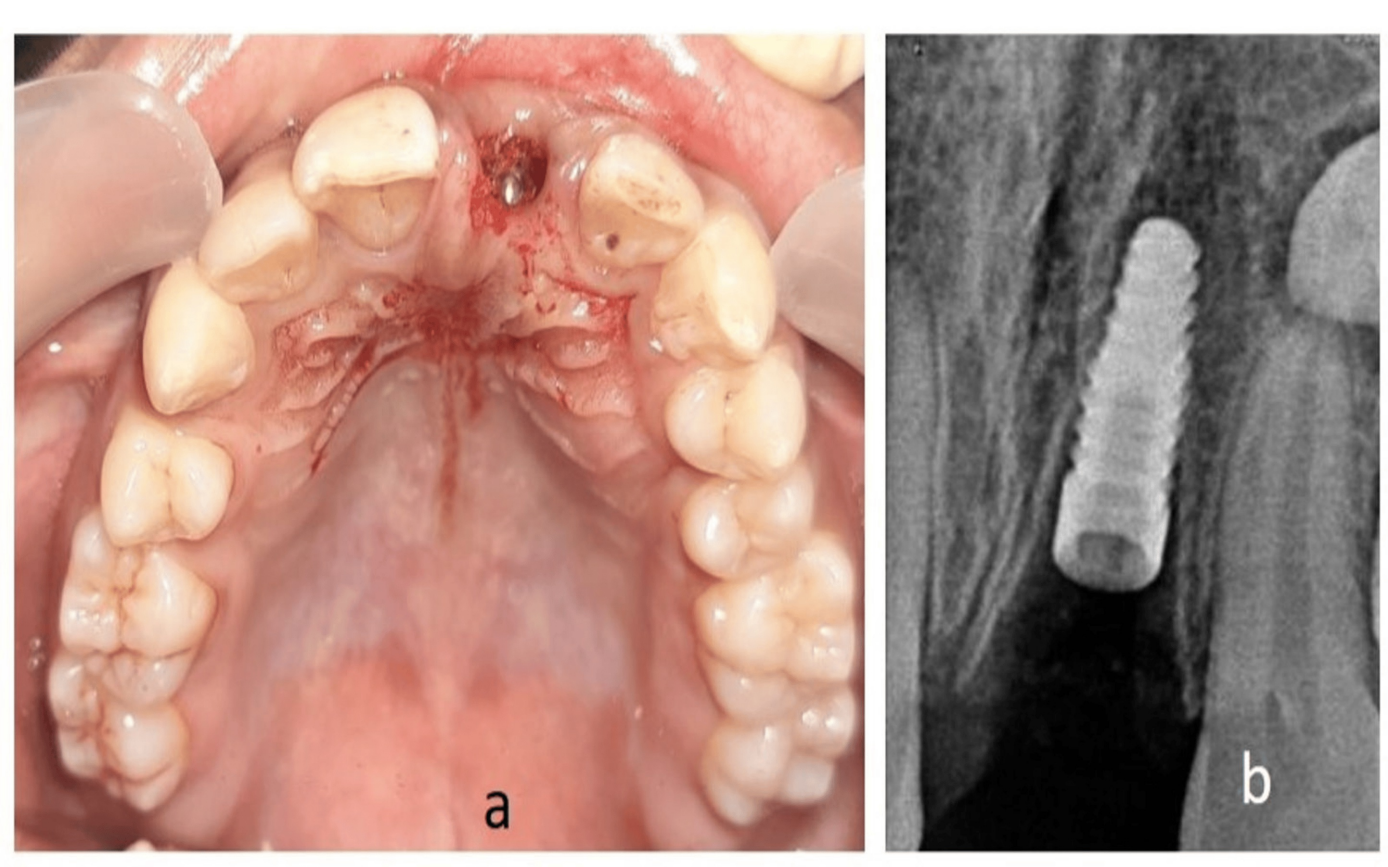
(a) Intraoral occlusal view showing implant placed; (b) Radiographic view of the implant

The pre-prosthetic treatment plan comprised of direct composite resin build-up of incisal edge for tooth no. 11. Prosthetic treatment included implant placement with tooth no. 21 followed by fabrication of customized healing abutment and temporization with fiber-reinforced resin-bonded prosthesis. A delayed loading protocol for final restoration (Type 2C-early implant placement + conventional loading) was decided to be followed. Two weeks post avulsion, after the soft tissue healing, a flapless surgery was performed. The osteotomy procedure was performed under local anesthesia. After verifying the position of the implant with the help of a parallel pin, a Biohorizon implant of diameter 3.8 mm and length 12 mm (BioHorizons Implant Systems Inc., Alabama, United States) was placed at the prepared site (Figure 4).

The implant was placed 3 mm apical to the cementoenamel junction of the adjacent tooth, and 1.5 mm from the root [21,22]. The fabrication of a customized healing abutment using bisacrylic material is crucial, aimed at preserving the dimensions of the extraction socket and supporting the soft tissues during the healing phase. To begin, the clinician carefully assessed the dimensions of the extraction socket and surrounding soft tissues. Using this information, a temporary abutment was selected and modified to closely approximate the shape and contour of the natural tooth. However, due to variations in socket morphology and soft tissue architecture, the prefabricated abutment was not an ideal fit.

**Figure 5:**
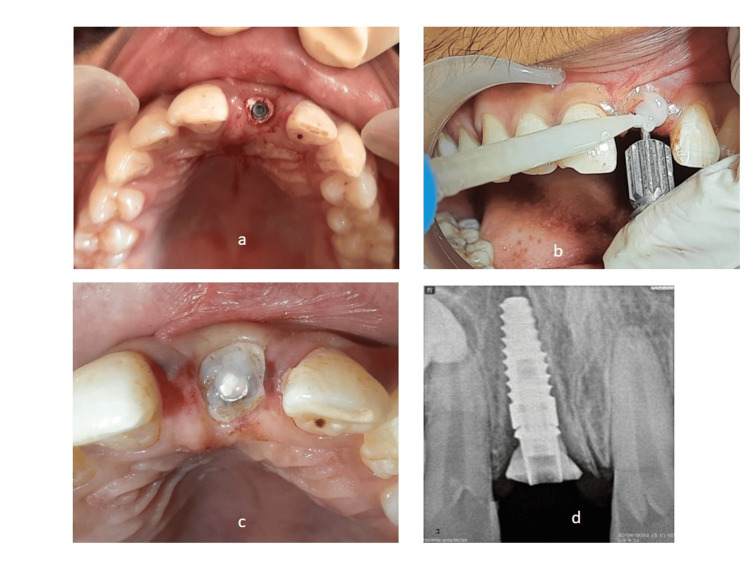
Fabrication of customized healing abutment (a) Space between the implant and soft tissue packed with Teflon; (b) Injecting bis-acrylic around abutment for customization; (c) Customized temporary abutment in place; (d) Radiographic evaluation to check complete seating of abutment

A customized healing abutment was fabricated using bisacrylic material. Bisacrylics offer several advantages in this context, including ease of manipulation, rapid polymerization, and biocompatibility [23]. These materials can be quickly molded and adapted to the specific contours of the socket and soft tissues, ensuring a snug fit and optimal support during the healing period. The fabrication process begins by loading the space between the temporary abutment and gingiva with Teflon tape or similar material to prevent the bis-acrylic resin from flowing onto the soft tissues. Next, the bis-acrylic material, Luxatemp Automix Plus (DMG Chemisch-Pharmazeutische Fabrik GmbH, Hamburg, Germany), was injected into the prepared space using a dispensing gun. Care was taken to ensure complete coverage of the temporary abutment and proper adaptation to the surrounding tissues. Once the material had polymerized, the temporary abutment was carefully removed and attached to an analog to facilitate further processing. The space between the abutment and the analog was then filled with composite resin to provide additional support and stability. The height of the healing abutment was adjusted as needed to ensure proper emergence profile and soft tissue support. Finally, the fabricated healing abutment was polished to a smooth finish to minimize plaque accumulation and irritation to the surrounding tissues. After verifying the fit and esthetics, the customized healing abutment was placed in the patient's mouth (Figure 5), where it served to maintain the dimensions of the extraction socket and support the overlying soft tissues during the critical healing phase following implant placement[24].

**Figure 6:**
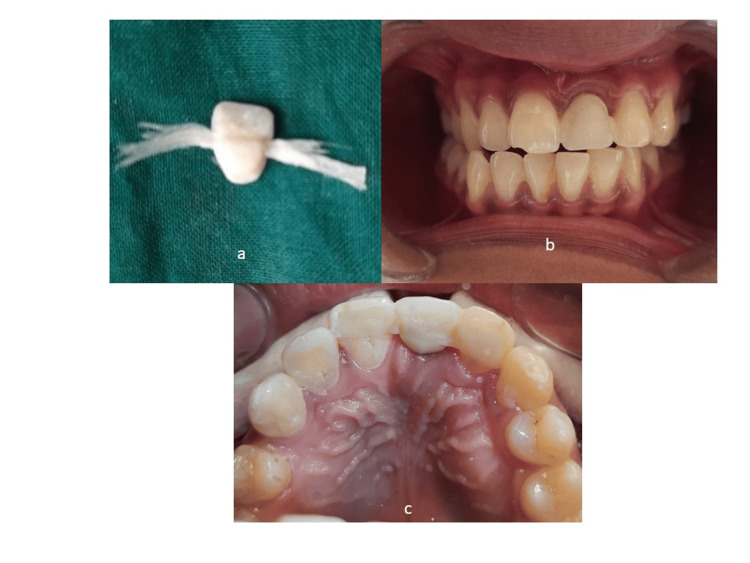
: Fabrication of fiber-reinforced resin-bonded temporary restoration. (a) Insertion of Interlig fibers through the channel made on the palatal surface of the acrylic tooth; (b) Labial view of the temporary restoration in situ; (c) Occlusal view of bonded temporary restoration

The method of temporization included placing an abutment followed by temporary restoration on the same day the fixture was placed. The use of a resin-bonded bridge, fiber-reinforced resin-bonded restoration, or a removable prosthesis has increased in order to maximize the pink esthetics around the anterior implant. Fiber-reinforced resin-bonded temporary restoration, which was independent of the implant, was then made and placed at the site (Figure 6). Preoperative cast was used to select the size and shape of the tooth. A tunnel was prepared above the cingulum through which fibers were passed. The gingival surface was further rounded off. Intraorally the adjacent teeth were etched, and Interlig fibers (Angelus Indústria de Produtos Odontológicos S/A, Londrina, Brazil), were bonded and cured. Occlusion was adjusted so that it does not interfere with protrusive and lateral movement.

**Figure 7:**
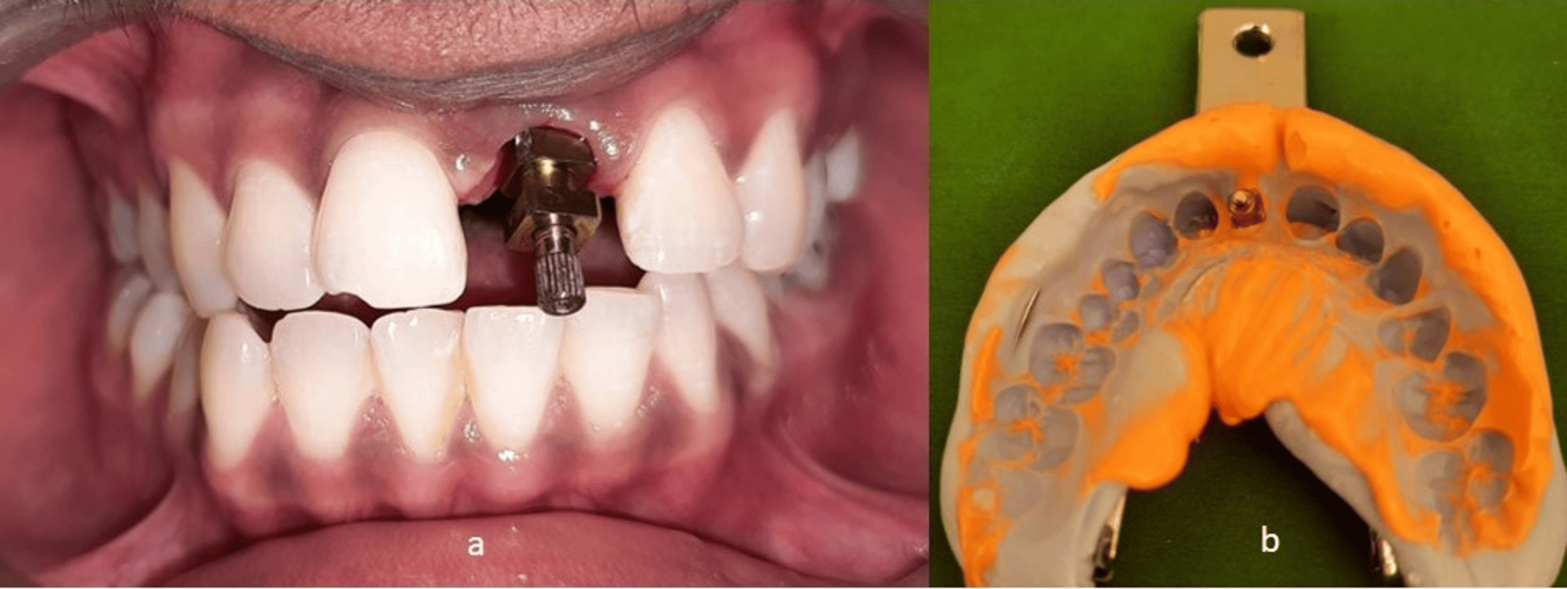
Open tray implant impression (a) Open tray impression coping placed; (b) Open tray impression made

The patient was instructed on proper brushing techniques with a small, soft bristle manual or electric toothbrush in a circular motion and massaged once a week with fingers for soft tissue maintenance to ensure optimal health during the implant healing phase. At follow-up visits in the first and third months, the patient reported that the fiber-reinforced resin-bonded restoration maintained its integrity and esthetic appearance. The prosthetic phase was initiated after the completion of four months. Implant-level impression was made using the open tray technique (Figure 7).

**Figure 8:**
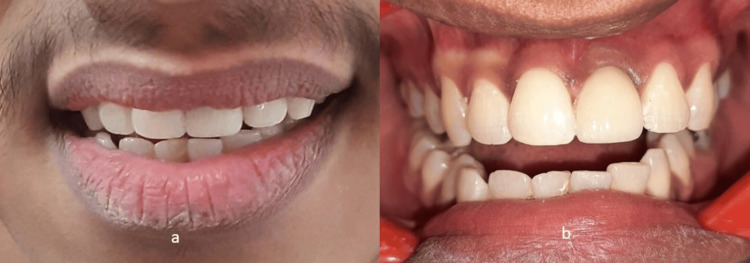
Cement retained PFM final prosthesis (a) Postoperative extraoral view; (b) Intraoral view showing final prosthesis in place PFM: porcelain fused to metal

**Figure 9:**
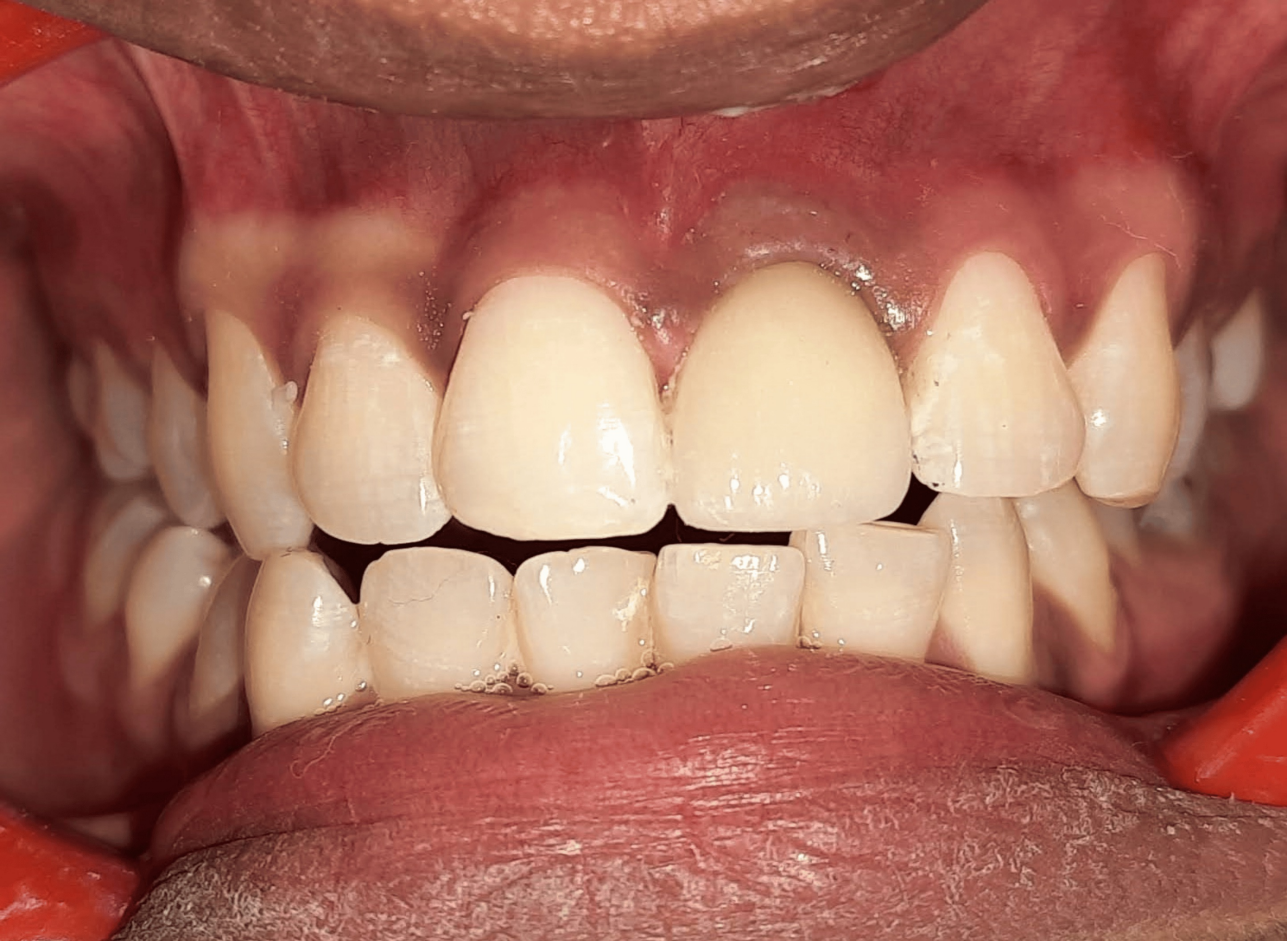
Final prosthesis in protrusion showing group function occlusion

This technique was selected as the length of the incisor was greater and the gingival depth was 3 mm. This was followed by the fabrication of smart (customized) abutment and shade selection. The seating of the abutment was verified by a radiograph. The abutment was given a torque of 15 N. Favorable implant angulation and optimal prosthetic space available enabled us to proceed with a cement-retained final prosthesis. A cement-retained porcelain fused to metal (PFM) crown was then cemented in the patient’s mouth after evaluating the form, function, and esthetics (Figure 8 and Figure 9). 

Throughout the treatment journey, patient education on oral hygiene and soft tissue maintenance played a crucial role in ensuring successful healing and long-term implant stability. Follow-up assessments demonstrated satisfactory comfort and restoration integrity, validating the effectiveness of the chosen approach.

## Discussion

Implants were initially placed in healed ridges. However, clinicians found it quite challenging to achieve favorable esthetics due to alveolar bone remodeling post extraction of the tooth. Patients were also concerned about the increased treatment time. Thus, early implant placement protocol proved to be a boon in implant dentistry. The goal of providing patients with early replacement of recently extracted teeth is to achieve the best possible soft-tissue esthetics, which is a desirable treatment objective [1]. Alternative methods are available to address the volumetric changes that occur after tooth extraction, such as site preservation and grafting procedures for both hard and soft tissues.

The case reported in this article illustrates how soft tissue esthetics was achieved by early placement of a dental implant and soft tissue modeling. An early implant placement was planned for this patient, after a thorough assessment of the Pink and White esthetics and considering the high esthetic demands of the patient. Implant placement with effective loading protocols is an important factor in implant treatment planning. Studies have revealed that immediate and early placement of the implant to replace a single missing tooth in the anterior maxilla offers parallel outcomes concerning dimensional ridge changes and achieves favorable esthetic, clinical, and patient-reported results [11]. Immediate implants show a higher incidence of midfacial recession than early implant placement, impacting around 26% of the sample [25]. In cases having thin or compromised facial walls also, early implant placement has shown a significant increase in the thickness of soft tissue. This also leads to the generation of a thick, soft tissue flap, enhancing vascularity and promoting superior healing. The risk of tearing of the flap and the requirement for soft tissue augmentation are also diminished. Early implant placement along with the added advantage of soft tissue healing provides benefits as it acquires 2-3 mm of extra keratinized mucosa around the implant leading to greater socket healing and apical bone formation [3,26].

This case report demonstrated excellent esthetic results and functional consequences with early implant placement. At the one-year follow-up, there was no vertical bone loss or soft tissue recession and there was a good amount of soft tissue surrounding the implant. Indeed, early implant placement is crucial in maintaining soft tissue health. Management of soft tissue plays a vital role in the final esthetic result as the form, color, and contour should be in harmony with the adjacent tissues.

In the late 1990s, a breakthrough emerged with this protocol known as Type 2 placement. This approach involves a healing period of four to six weeks or 12-16 weeks post extraction before implant placement. During this critical phase, a series of advantageous biological processes occur, which simplify the surgical procedure and minimize the likelihood of post-surgical complications [12]. Osseointegration of a dental implant after placement follows stages of repair. The first stage involves the initial formation of a blood clot at the implant site, triggered by the surgical trauma. This blood clot serves as a scaffold for subsequent tissue regeneration processes. In the second stage, cellular activation occurs within the blood clot. Components of the blood, such as platelets and growth factors, interact with the surface of the implant, leading to the adsorption of fibrin on the implant surface. This fibrin network acts as a framework for cellular migration and attachment, facilitating the subsequent stages of tissue repair. The third stage includes cellular responses for osseointegration. Bone cells, including osteoblasts and osteoclasts, migrate into the blood clot and initiate the process of bone formation. Osteoblasts deposit osteoid matrix, which subsequently undergoes mineralization to form new bone tissue. Osteoclasts are involved in the resorption of bone fragments and remodeling of the bone tissue to accommodate the implant [4]. These processes culminate in the establishment of osseointegration, ensuring the stability and functionality of the implant in the oral environment.

A literature search was conducted in the PubMed database for systematic reviews and meta-analysis including esthetic and functional outcomes for implants loaded with early, immediate, and delayed implants.

In studies, by Bassir et al. [11], Chen and Buser [12], and Esposito et al. [27], quantitative analyses were performed to evaluate the efficacy of immediate, early placement protocols and conventionally or delayed loaded implants, particularly in the esthetic zone. The primary outcome variable was failed implants. Additionally, effectiveness and safety loading protocol of dental implants, particularly with flapless placement techniques. The findings collectively indicate no significant difference in survival and success rates between early and immediate protocols. This suggests that both protocols yield comparable outcomes in terms of implant survival and success, providing valuable insights for clinical practice.

Good primary stability is crucial for achieving desirable results in immediate and early placement protocols. Studies also included the evaluation of esthetic outcomes to determine the optimal timing for implant placement using different protocols and parameters. Sanz et al. concluded that early placement favored a lower percentage of bone height and bone width reduction [28]. Esposito et al. reported no statistically significant differences for prosthesis failures and marginal bone loss on an individual patient basis [29]. Riachi et al. found that immediate implants had significantly superior Pink Esthetic Score (PES), marginal bone loss, and plaque index results compared to early implants, despite having a higher probing depth in the immediate group [30]. Bassir et al. suggested that early implant placement protocols show similar outcomes to other placement protocols [11]. Ickroth et al. noted no significant differences in esthetic and clinical outcomes between immediate and early implant placement in low-risk patients with an intact buccal bone wall, highlighting the importance of stability of peri-implant hard tissue [31].

Chen et al. also included post-extraction sites and the influence of simultaneous bone augmentation procedures on outcomes, suggesting acceptable esthetic outcomes [32]. Asghar et al. reported similar sentiments, indicating comparable esthetics and clinical results between immediate and delayed placement protocols [33]. Additionally, factors such as patient discomfort and chair time favored early and immediate protocols due to their faster, time-saving, and flapless techniques. These evidences are taken into consideration for placing early implant protocol for this case to achieve optimum esthetic. This also offers several benefits for the surgeon from enhanced vascularity within the flap, which improves healing capacity, attributed to the presence of a thick mucoperiosteal flap for implant surgery. It also reduces the need for connective tissue grafting for augmentation [2]. Any infections at the extraction site will resolve presenting a potential implant site with diminished bacterial risk. The socket's apical portion exhibits newly formed bone which enables effortless implant bed preparation.

Pow and McMillan first introduced customized healing abutments with the idea of creating a natural-looking appearance around the implant [34]. According to the above technique, retentive grooves were made on the surface of standard healing abutment. Following this, the resin is added to mimic the life-like gingival contour of the final prosthesis, eliminating the requirement for previsualization. Follow-up after two weeks of insertion revealed natural soft tissue contour. Incorporating the above technique of healing abutment in implant dentistry ensures a desirable soft tissue contour.

There are various studies that have incorporated the idea of custom healing abutment using chairside fabrication [35]. The healing abutment can be customized using the direct method or indirect method in the immediate placement protocol. In the direct method, the temporary cylinders are connected to the implant fixture, after which composite resin is added to create the outline of the tooth. The indirect method includes making an impression conventionally or digitally and then fabricating it with the computer-aided design/computer-aided manufacturing (CAD/CAM) technique. The main idea is to create an emergence profile along with the closure of the implant site.

In delayed implant placement, there is no tooth present hence fabrication of this custom-made abutment helps us to achieve the required contour of final restoration during implant placement. It can be done by taking an impression prior to placing an implant and fabricating it with the help of a diagnostic cast, or advanced implant planning software along with milling materials. According to the literature, customized abutments and custom posts yield better transmucosal contour and have better soft tissue response. It has also led to promising outcomes in terms of soft tissue contour and emergence profile [36].

The technique used in this case report of fabricating customized healing abutment directs an alternative treatment where the second surgical stage is avoided, which is necessary to expose the implant while maintaining the natural contour of soft tissues. In conventional technique, second-stage surgery is performed as the start of the prosthetic part of the treatment. The implant is exposed followed by the placement of a healing cap. Immediate insertion of customized healing abutment prevents the second stage surgery and has advantages such as spontaneous soft tissue healing, ensuring an additional 3-5 mm of keratinized mucosa in the prospective implant area and in areas characterized by a thin facial bone wall phenotype, or where the facial wall has been compromised, there will be spontaneous thickening of the soft tissue [37].

For optimal esthetic outcomes with implant restorations, it is essential to modify the peri-implant soft tissue during the provisional stage, creating a suitable emergence profile and natural contour. In this case report the above was achieved with the help of a customized healing abutment made up of bis-acrylic. Customized temporary abutment promotes the establishment of desired gingival architecture and eliminates the requirement for a removable prosthesis throughout the healing period [37].

The final restoration was made using a smart (customized) abutment. The above technique ensures accurate fit, decreases procedural expenses, and eliminates dimensional inaccuracies attributed to conventional waxing and casting techniques. The longevity of the implant and prosthetic success is enhanced due to the accurately fitting custom implant abutment and thus the restoration process is streamlined [38]. Digital technology enables clinicians to customize the implant abutment and create natural-looking superstructures that harmonize with the patient's dentition [39].

This case report underscores the significance of a multidisciplinary careful patient evaluation and customized treatment planning in achieving desirable outcomes, particularly in challenging esthetic scenarios. By addressing patient concerns and leveraging advanced techniques, successful implant rehabilitation can be achieved, restoring both function and esthetics to enhance patient quality of life.

## Conclusions

This case report highlights the importance of customized treatment planning and precise execution for a missing upper anterior tooth due to trauma, especially in esthetically sensitive areas. The patient, initially reluctant toward conventional prosthetics, received a meticulously crafted dental implant strategy to meet cosmetic expectations. Thorough evaluation of detailed esthetic assessments guided the treatment plan, ensuring a harmonious integration of Pink and White esthetics. The approach included flapless implant surgery with a delayed loading protocol to preserve soft tissue contours and promote optimal healing. A customized healing abutment was used to maintain socket dimensions during healing, while immediate temporary restoration post-implant enhanced patient comfort and esthetics. Follow-up assessments confirmed satisfactory comfort levels and preservation of restoration integrity, affirming the chosen approach's efficacy.

Early implant placement offers significant advantages, such as reduced surgical intervention, enhanced soft tissue healing, and maintenance of gingival architecture. This case was suitable due to intact bone plates and favorable soft tissue healing, contributing to initial stability and esthetic outcomes. Custom implant abutments played a crucial role in enhancing implant emergence profiles, essential for long-term esthetic success. Anatomically shaped customized cervical margins aligned with natural tooth roots and compensated for implant angulation issues. Patient education on oral hygiene and soft tissue maintenance was vital for successful healing and long-term stability. Dental implant placement followed by crown restoration aims to deliver durable and pleasing solutions while preserving adjacent teeth. Collaboration between the patient and dental team is essential throughout the treatment journey to ensure optimal outcomes and patient satisfaction.
